# Improvement of Obesity and Dyslipidemic Activity of *Amomum tsao-ko* in C57BL/6 Mice Fed a High-Carbohydrate Diet

**DOI:** 10.3390/molecules26061638

**Published:** 2021-03-15

**Authors:** Ju-Hyoung Park, Eun-Kyung Ahn, Min Hee Hwang, Young Jin Park, Young-Rak Cho, Hye-Jin Ko, Wonsik Jeong, Seung Hwan Yang, Dong-Wan Seo, Joa Sub Oh

**Affiliations:** 1 College of Pharmacy, Dankook University, Dandae-ro 119, Dongnam, Cheonan, Chungnam 31116, Korea; yourselves@naver.com (J.-H.P.); dwseomb@dankook.ac.kr (D.-W.S.); 2 Bio-Center, Gyeonggido Business and Science Accelerator, Gwanggyo-ro 147, Yeoungtong, Suwon, Gyeonggi 16229, Korea; aek@gbsa.or.kr (E.-K.A.); mhhwang@gbsa.or.kr (M.H.H.); yjp@gbsa.or.kr (Y.J.P.); yrcho@gbsa.or.kr (Y.-R.C.); ko114j@gbsa.or.kr (H.-J.K.); ws2009@gbsa.or.kr (W.J.); 3 Department of Biotechnology, Chonnam National University, Yeosu, Chonnam 59626, Korea; ymichigan@chonnam.ac.kr

**Keywords:** antiobesity, antidyslipidemia, *Amomum tsao-ko*, liver tissue, high-carbohydrate diet

## Abstract

*Amomum tsao-ko* Crevost et Lemaire (Zingiberaceae) is a medicinal herb found in Southeast Asia that is used for the treatment of malaria, abdominal pain, dyspepsia, etc. The aim of this study was to investigate the effect of an ethanol extract of *Amomum tsao-ko* (EAT) on obesity and hyperlipidemia in C57BL/6 mice fed a high-carbohydrate diet (HCD). First, the mice were divided into five groups (*n* = 6/group) as follows: normal diet, HCD, and HCD+EAT (100, 200, and 400 mg/kg/day), which were orally administered with EAT daily for 84 days. Using microcomputed tomography (micro-CT) analysis, we found that EAT inhibited not only body-weight gain, but also visceral fat and subcutaneous fat accumulation. Histological analysis confirmed that EAT decreased the size of fat tissues. EAT consistently improved various indices, including plasma levels of total cholesterol (TC), triglyceride (TG), low-density lipoprotein, high-density lipoprotein, atherogenic index, and cardiac risk factors, which are related to dyslipidemia—a major risk factor for heart disease. The contents of TC and TG, as well as the lipid droplets of HCD-induced hepatic accumulation in the liver tissue, were suppressed by EAT. Taken together, these findings suggest the possibility of developing EAT as a therapeutic agent for improving HCD-induced obesity and hyperlipidemia.

## 1. Introduction

The consumption of high-calorie foods affects lipid metabolism and leads to fat accumulation resulting in obesity, which is a cause of various metabolic diseases such as hyperlipidemia, atherosclerotic disease, and cardiovascular disease. The prevalence of these metabolic diseases caused by a high-calorie diet has increased steadily in recent decades [1,2,3].

Hyperlipidemia, characterized by a high lipid profile in the blood owing to an abnormality in lipid metabolism, is strongly related to obesity and is known to be a major risk factor for cardiovascular disease [4,5]. Obesity is defined as an accumulation of excessive body fat due to overnutrition and reduction in energy expenditure, and is related to lipid accumulation in adipocytes and the liver, resulting in adipocyte hyperplasia and hypertrophy [5,6,7]. These processes are responsible for obesity-related metabolic diseases such as dyslipidemia, in which total cholesterol (TC), triglyceride (TG), high-density lipoprotein (HDL), and low-density lipoprotein (LDL) are important characteristics [8,9]. In particular, the liver is an important organ in charge of lipid metabolism within fatty tissue. Free fatty acid is fundamental to the body’s energy metabolism, and is stored in the form of triglyceride and transferred to other organs. When an imbalance between inflow and outflow of triglyceride occurs in these lipid metabolism processes, an accumulation of triglyceride occurs in tissue [10,11]. Since excessive lipid absorption and abnormal lipid metabolism cause obesity-related metabolic diseases, it is necessary to improve the lipid profile of the blood to prevent disease. Further, adipogenesis is modulated by gene expression, which induces the differentiation of adipocytes regulated by signaling cascades involving transcription factors such as the CCAAT-enhancer-binding-protein α (C/EBP-α) and peroxisome proliferator-activated receptor γ (PPAR-γ). C/EBP-α and PPAR-γ play a major role in the differentiation of adipocytes by independently or cooperatively mediating their differentiation into mature adipocytes in the liver [12,13,14].

Numerous chemical and natural agents have been shown to treat hyperlipidemia. Many chemical drugs, such as statins, decrease plasma cholesterol but have various side effects. Therefore, dietary habits should be improved, alongside the consumption of functional foods or drugs that improve the plasma lipid profile, to prevent hyperlipidemia [15,16].

*Amomum tsao-ko* Crevost et Lemaire (*A. tsao-ko*), belonging to the Zingiberaceae family, is distributed naturally in Southeast Asia. *A. tsao-ko*, commonly used as food and as a spice, is used in traditional medicine to treat digestive and stomach disorders [17,18,19]. It has been reported that *A. tsao-ko* reduces the inflammatory response induced by lipopolysaccharides, and various biologically active substances from *A. tsao-ko* are known to be effective antioxidant, antimicrobial, and antiobesity agents that can treat several diseases [20,21,22]. However, the ability of *A. tsao-ko* to improve the lipid profile and reduce lipid accumulation in C57BL/6 mice fed a high-carbohydrate diet (HCD) has not yet been reported in vivo.

Therefore, the aim of this study was to investigate the antidyslipidemic effects of an ethanol extract of *A. tsao-ko* (EAT), focusing on the improvement of plasma lipid profiles and the reduction in the accumulation of adipocytes in vivo.

## 2. Results

### 2.1. Effects of EAT on Body Weight and Food Intake

To observe the effects of EAT in an HCD-induced mouse model, C57BL/6 mice were fed an HCD with EAT (100, 200, and 400 mg/kg/day), orally administered for 12 weeks. The ingredients of the HCD are presented in Table 1. There was little difference in the initial body weight, but body weight in the HCD group increased compared with the normal diet (ND) group by the end of the experiment. Compared with the HCD group, weight loss was seen in all of the EAT treatment groups (Table 2). In addition, there was no difference in food intake among the HCD groups after 12 weeks (Table 3).

### 2.2. Effect of EAT on Quantification of Fat Depot

**Figure 1:**
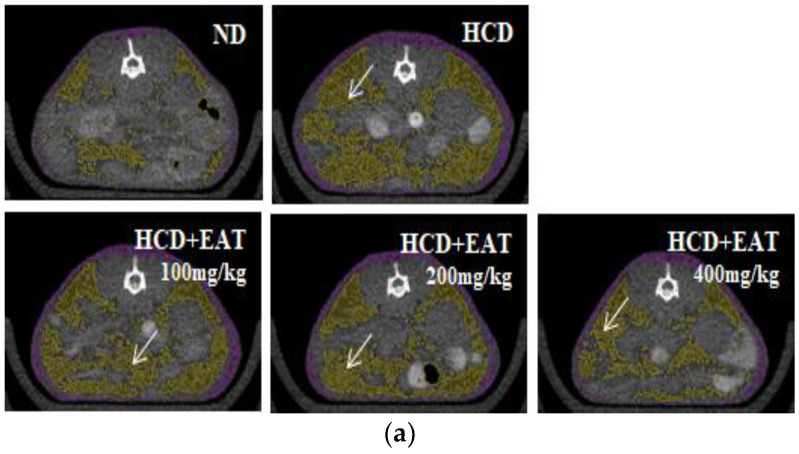

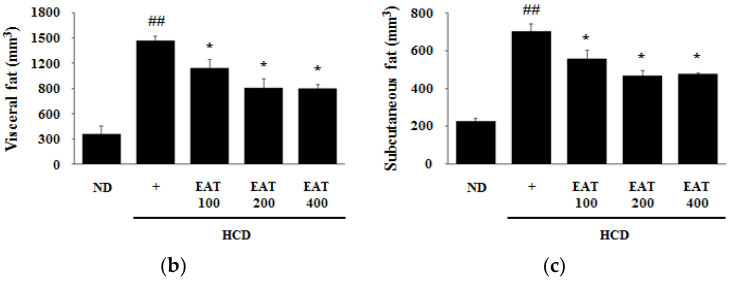
Effect of EAT on the change of body fat profile in an HCD-induced C57BL/6 mouse model. (**a**–**c**) To assess the change of fat accumulation induced by HCD, mice were anesthetized and (**a**) body fat accumulation, including (**b**) visceral and (**c**) subcutaneous fat, was measured by microcomputed tomography (micro-CT) at the end of experiment. Values are expressed as mean ± SD; ^##^ *p* < 0.01 compared with the ND group; * *p* < 0.05 compared with the HCD group.

To evaluate the quantification of fat depot, a microcomputed tomography (micro-CT) analysis was performed. Figure 1a shows a micro-CT image of the abdominal fat accumulation that was used to evaluate the effect of EAT on body fat profile. The amounts of visceral fat and subcutaneous fat were increased in the HCD group compared with their levels in the ND group. EAT treatment significantly decreased the volume of visceral and subcutaneous fat in all treatment groups, and the 200 and 400 mg/kg EAT-treated groups showed similar reductions in visceral and subcutaneous fat (Figure 1b,c).

### 2.3. Effects of EAT on Changes in Weight and Morphology of White Adipose Tissue

**Figure 2:**
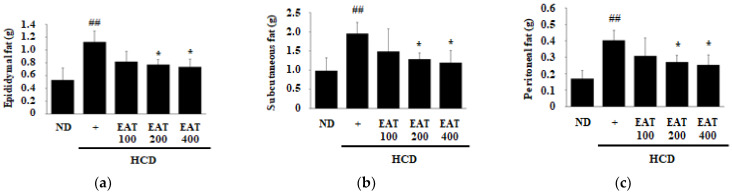

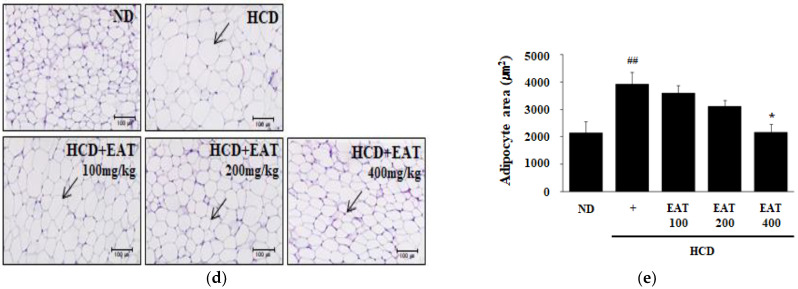
Effect of EAT on the white adipose tissue (WAT) weight and histological analysis in an HCD-induced C57BL/6 mouse model. (**a**–**c**) To assess the change in body weight induced by HCD, WAT was collected and the weight for each area including (**a**) epididymal, (**b**) subcutaneous, and (**c**) peritoneal fat was immediately measured at the end of the experiment. (**d**,**e**) For histological analysis of WAT to analyze adipocyte size, WAT was collected and fixed in 10% neutral buffered formalin. The embedded paraffin blocks were cut into 5 μm sections and stained with hematoxylin and eosin (H&E). (**d**) The change in size of adipocytes was observed under a light microscope at 100× magnification and (**e**) the quantification of adipocyte area was measured by ImageJ software. Values are expressed as mean ± SD; ^##^ *p* < 0.01 compared with the ND group; * *p* < 0.05 compared with the HCD group.

It has been reported that HCD affects weight gain and the morphology of white adipose tissue (WAT) [23]. Therefore, the regulatory effect of EAT on the weight of WAT, epididymal, subcutaneous, and peritoneal adipose tissue was measured (Figure 2a–c). The relative weight of all adipose tissues was increased in the HCD group compared with the ND group, and, compared with the HCD group, the weight of all adipose tissues decreased in the EAT treatment groups. Compared with the HCD group, 200 and 400 mg/kg EAT-treated groups demonstrated significantly decreased adipose tissue weight in all WAT. In order to measure the effect of EAT on morphological changes and to confirm the adipose mass in the subcutaneous fat, we performed histological photography and adipocyte area analysis. As shown in Figure 2d, the adipocyte size in the HCD group was larger than that of the ND group. All of the EAT treatment groups showed a reduction in the adipocyte size in a dose-dependent manner compared with the HCD group (Figure 2e).

### 2.4. Effects of EAT on Plasma Lipid Parameters

**Figure 3:**
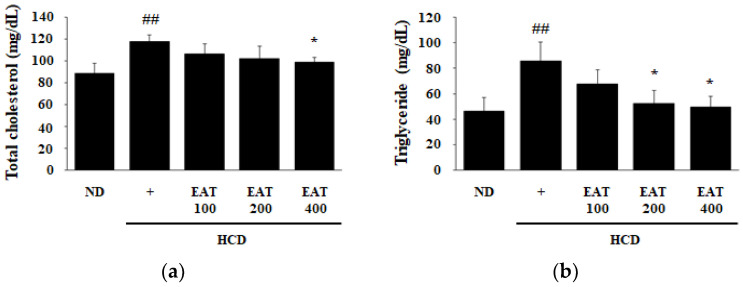

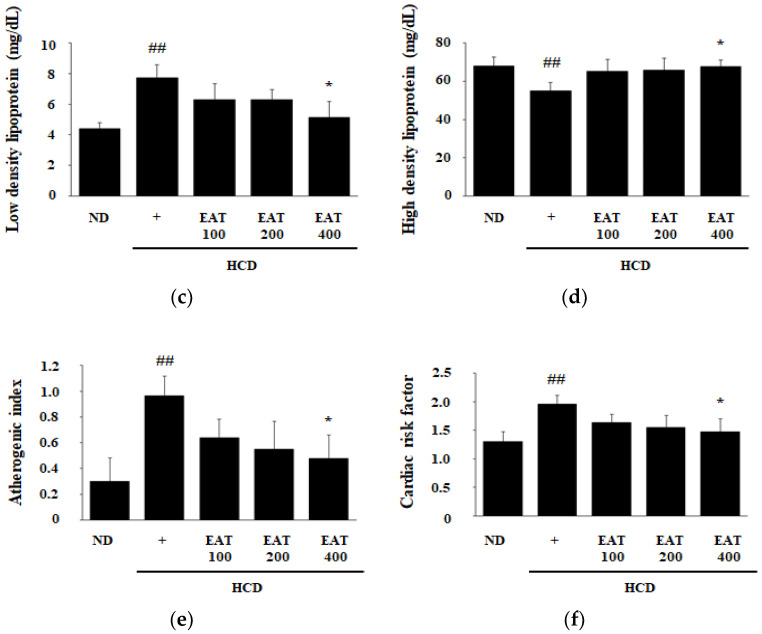
Effect of EAT on the plasma lipid profile and cardiovascular disease risk in an HCD-induced C57BL/6 mouse model. (**a**–**d**) Blood was collected for centrifugation at 3000 rpm for 15 min at 4 °C to separate plasma for analysis of the change in plasma lipid profile at the end of the experiment. Plasma lipid profile including (**a**) total cholesterol (TC), (**b**) triglyceride (TG), (**c**) low-density lipoprotein (LDL), and (**d**) high-density lipoprotein (HDL) was analyzed by biochemical analyzer. (**e**,**f**) The risk index of cardiovascular disease determined as (**e**) atherogenic index (AI) and (**f**) cardiac risk factor (CRF) was based on TC and HDL. AI = (TC − HDL)/HDL, CRF = TC/HDL. Values are expressed as mean ± SD; ^##^ *p* < 0.01 compared with the ND group; * *p* < 0.05 compared with the HCD group.

Dyslipidemia, due to a diet containing a large amount of fat or abnormal lipid metabolism, occurs when lipids including TC, TG, and LDL are higher than normal or when HDL is lower than normal in the blood [24]. Therefore, to analyze the effect of EAT in an HCD-induced mouse model, the plasma lipid parameters TC, TG, LDL, and HDL were measured. As shown in Figure 3a–d, the TC, TG, and LDL levels in the HCD group were increased when compared with the ND group, and HDL level in the HCD group was decreased compared with the ND group. In the treatment groups, the levels of TC and TG were reduced in a dose-dependent manner compared with the HCD group (Figure 3a,b). In the 400 mg/kg EAT-treated group, the levels of TC, TG, and LDL were significantly reduced and the level of HDL was increased, compared with the HCD group. The TG level in the 200 mg/kg EAT-treated group was markedly reduced compared with the HCD group. To determine the effect of EAT on the risk of hyperlipidemia, we calculated the atherogenic index (AI) and cardiac risk factor (CRF) values. The AI and CRF values were decreased in EAT-treated groups, especially in the 400 mg/kg EAT-treated group, compared with levels in the HCD group (Figure 3e,f).

### 2.5. Effects of EAT on Lipid Accumulation and TC and TG Content in Liver Tissue

**Figure 4:**
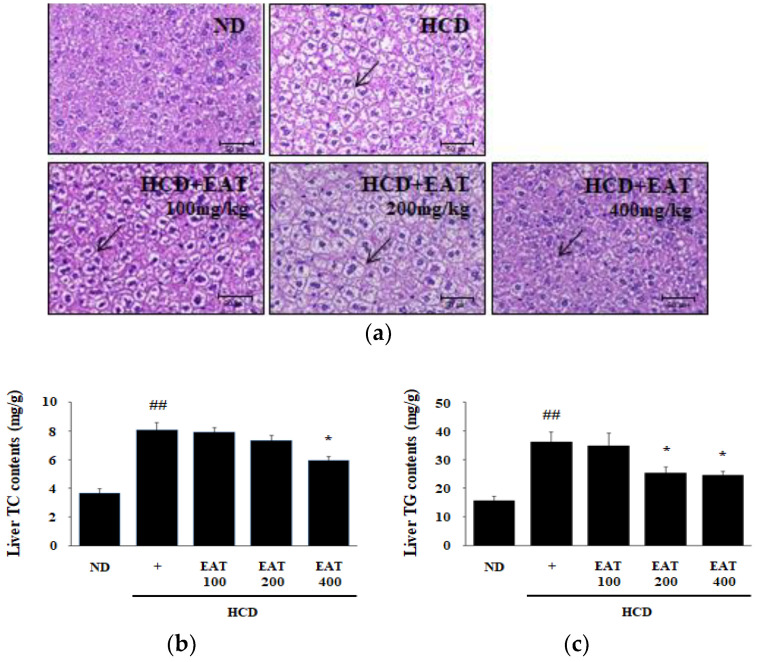
Effect of EAT on hepatic histological analysis and function in an HCD-induced C57BL/6 mouse model. (**a**–**c**) To assess the change induced by HCD, liver was collected and (**a**) histologically analyzed to detect lipid accumulation. Liver was fixed in 10% neutral buffered formalin and embedded in paraffin. The paraffin blocks were cut into 5 μm sections and stained with H&E. The accumulation of fat globules in liver was observed under a light microscope at 200× magnification (**b**,**c**). To assess liver function, liver lipid was extracted. The hepatic function determined as (**b**) TC and (**c**) TG contents was measured. Values are expressed as mean ± SD; ^##^ *p* < 0.01 compared with the ND group; * *p* < 0.05 compared with the HCD group.

It has been reported that HCD affects the hypertrophy of adipose tissue and lipid accumulation in the liver. Lipid accumulation in the liver is associated with lipid metabolism leading to hepatic steatosis. The degree of hepatic steatosis is measured by the concentration of TC and TG contents in liver tissue [25,26,27]. By the formation of lipid droplets in the liver, the accumulation of fat globules increased in the HCD group compared with the ND group. In all EAT treatment groups, hepatic lipid droplets were attenuated compared with the HCD group (Figure 4a). In addition, EAT treatment decreased the levels of TC and TG in the liver tissue. Liver TC content in the 400 mg/kg EAT-treated group was reduced, and liver TG contents in the 200 and 400 mg/kg EAT-treated groups were markedly reduced. This result was similar to the plasma lipid profile in HCD-fed mice (Figure 4b,c).

### 2.6. Effect of EAT on Adipocyte-Differentiation-Related Protein Levels of C/EBP-α and PPAR-γ

**Figure 5:**
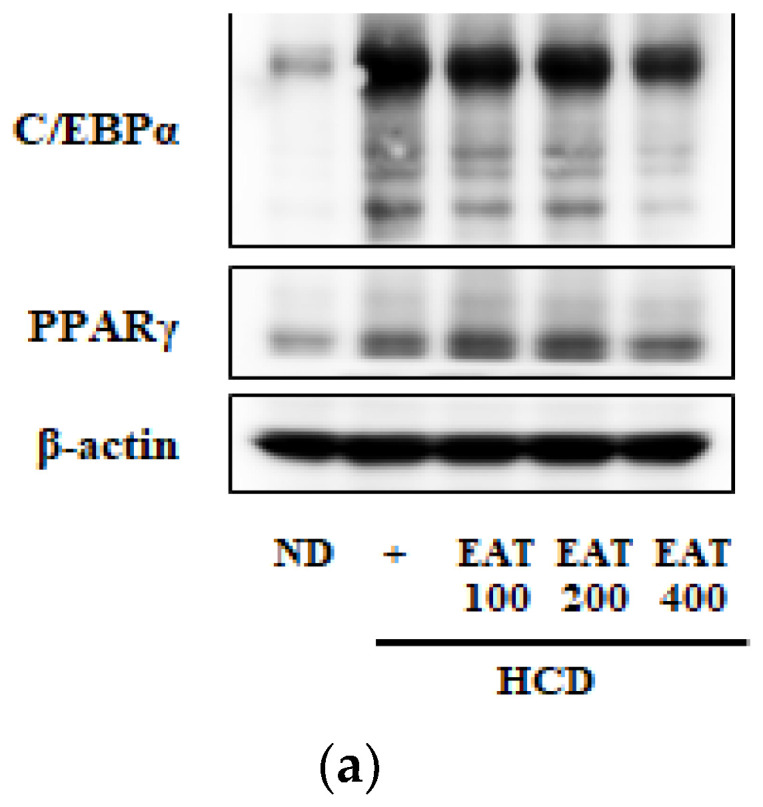

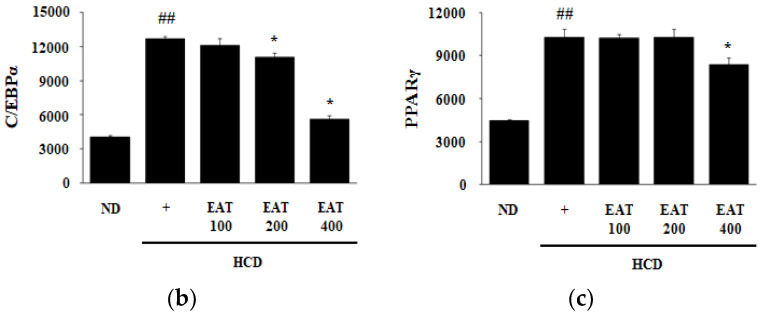
Effect of EAT on protein expression of PPAR-γ and CEBP-α in an HCD-induced C57BL/6 mouse model. (**a**–**c**) To measure the adipogenesis of liver induced by HCD, the expression of PPAR-γ and CEBP-α was determined by Western blot and the representation of PPAR-γ and CEBP-α was analyzed by densitometry protocol. Values are expressed as mean ± SD; ^##^ *p* < 0.01 compared with the ND group; * *p* < 0.05 compared with the HCD group.

To investigate the effect of EAT on adipocyte differentiation, the protein expression of adipocyte-differentiation-related transcription factors C/EBP-α and PPAR-γ was measured by Western blot analysis. As shown in Figure 5, the protein expression level of C/EBP-α and PPAR-γ was increased in the HCD group compared with the ND group. In all EAT treatment groups, the protein expression of C/EBP-α and PPAR-γ was inhibited compared with the HCD group, especially in the 400 mg/kg EAT-treated group.

## 3. Discussion

The *A. tsao-ko* used in this study belongs to the herb family Zingiberaceae; dried *A. tsao-ko* is a medicinal plant used in the treatment of various diseases [18]. Previous studies reported that extracts of *A. tsao-ko* were shown to be effective in oxidation inhibition, anti-inflammation, and regulation of liver lipids [19,20], but there are few studies on the associated antiobesity and antihyperlipidemic effects.

In order to confirm these in vitro findings in vivo, we examined the effect of EAT on obesity and dyslipidemia using an HCD-fed mouse model. In this study, it was demonstrated that EAT modulated obesity and its association with dyslipidemia in an in vivo experiment, which is in agreement with the existing in vitro data. After 12 weeks, compared with the HCD group, all of the groups treated with EAT showed a reduction in body-weight gain. Especially, 200 mg/kg and 400 mg/kg EAT treated-groups showed similar effects in visceral, subcutaneous, epididymal, and peritoneal fat condition, except the size of the adipocyte area. It was confirmed that EAT regulated obesity induced by an HCD (Table 2, Figure 1 and Figure 2). In particular, the reduction in visceral fat known to be the cause of various metabolic diseases was also confirmed [28]. Due to changes in dietary habits, the occurrence of obesity has increased, and one metabolic diseases caused by obesity is dyslipidemia. Consuming excess fat increases lipid deposition and causes the lipid profile of the vascular walls to worsen. Consequently, diseases, including cardiovascular disease, atherosclerosis, insulin resistance, and diabetes, occur in patients with dyslipidemia accompanied by obesity [2,10,29]. In the present study, we found that EAT treatment improved dyslipidemia factors such as plasma levels of TC, TG, LDL, and HDL and the AI and CRF (Figure 4). The consumption of HCD induced liver dysfunction by the accumulation of hepatic lipids, and also caused other problems related to obesity-induced dyslipidemia. Hepatic lipids, including TC and TG, are deposited in the liver, leading to a fatty liver [30,31]. The amounts of TC, TG, and lipid droplets associated with HCD-induced hepatic accumulation in the liver tissue were decreased by treatment with EAT (Figure 4). The differentiation of adipocytes is primarily regulated by various transcriptional signaling pathways. PPAR-γ and C/EBP-α are important adipogenic transcription factors which coregulate the differentiation and maturation of adipocytes. These transcription factors maintain the adipogenesis-induced expression of adipocyte-specific genes [32,33]. We demonstrated that EAT reduced adipogenesis by downregulating the protein level of C/EBP-α and PPAR-γ in the liver (Figure 5).

Our previous study reported the isolation of amotsaokonal A–C, benzaldehyde, and cycloterpenal, as new compounds extracted from EAT. We also isolated trans-nerolidol, a major active substance contained in EAT [19], and suggest further research on the various active components contained in EAT. Additionally, the molecular mechanisms and signaling pathways affected by EAT in response to antiobesity and antidyslipidemia using tissues needs further study. It is also necessary to investigate the effect of EAT on obesity and lipid parameters using a a high-fat diet animal model in future research. Nonetheless, the present study showed that the antiobesity effect of EAT seen in vitro is also exhibited in vivo, which provides support for the possibility of its use in a clinical trial.

In conclusion, these findings demonstrate, for the first time, that EAT improved HCD-induced obesity and dyslipidemia by inhibiting body-weight gain, visceral fat mass, and the levels of important indicators associated with dyslipidemia. These results suggest that pharmacological studies of EAT should be performed, and it is possible that the findings of these studies could contribute to the treatment of obesity or dyslipidemia in the future.

## 4. Materials and Methods

### 4.1. Plant

Dried *A. tsao-ko* was purchased from the Kyungdong Oriental Herbal Market in Korea (August 2016) and identified by one of the authors (Joa Sub Oh). A voucher specimen (G47) was deposited at the Natural Products Research Laboratory, Gyeonggido Business and Science Accelerator. The dried fruits *A. tsao-ko* (5 kg) were extracted with 80% ethanol (54 L) at room temperature for 48 h, and the extract was subsequently paper-filtered, concentrated by vacuum evaporation under reduced pressure, and centrifuged in a vacuum using a rotary evaporator. The resulting extract was completely freeze-dried to obtain a powder.

### 4.2. Animals and Experimental Design

Five-week-old male C57BL/6N mice were obtained from Orient, Inc. (Seoul, Korea) and housed in polycarbonate cages under standard conditions of 12 h light–dark cycle, 18–22 °C temperature, and 35–55% humidity in a specific-pathogen-free environment. All mice had free access to food and water for 1 week. After the acclimation of 1 week, all mice were fed with the ND (3.1 kcal/g, Teklad global 18% protein rodent diet, Envigo, Madison, WI, USA) or HCD (4 kcal/g, 70% carbohydrate, Envigo, Madison, WI, USA) ad libitum and were divided into five groups (*n* = 6/group) as follows: ND, HCD, HCD+EAT (100, 200, and 400 mg/kg/day). *A. tsao-ko* dissolved in phosphate-buffered saline (PBS) was orally administered at 100, 200, and 400 mg/kg and the HCD group was administered PBS without EAT once daily for 12 weeks. During the experiment, body weight and food intake were recorded twice a week. In compliance with the Guide for the Care and Use of Laboratory Animals, all experimental procedures were approved by the Institutional Animal Care and Ethical Use Committee of Bio-Center, Gyeonggido Business and Science Accelerator (2019-03-0001).

### 4.3. Biochemical Analysis of Blood

At the end of the experiment, all mice were anesthetized after fasting with isoflurane (HanaPharm, Seoul, Korea). Blood collected from the abdominal vena cava was rolled and immediately centrifuged at 3000 rpm for 15 min at 4 °C to collect plasma for blood biochemical analysis. The plasma samples were stored at −80 °C until analysis. The plasma levels of TC, TG, LDL, and HDL were analyzed using a biochemical analyzer (Hitachi 7020, Tokyo, Japan). The AI and CRF values were calculated based on the plasma lipid profile (TC and HDL).

### 4.4. Histological Analysis of Liver and Adipose Tissue

At the end of the experiment, the liver and WAT were collected and the weight of WAT for each area was measured. For histological analysis, the liver and WAT were fixed in 10% neutral buffered formalin and embedded in paraffin. The embedded paraffin blocks were cut into 5 μm sections and stained with hematoxylin and eosin (H&E) to observe the lipid accumulation and adipocyte size under light microscope (Nihon Kohden, Tokyo, Japan). To quantify adipocytes, the size and area of adipocytes were randomly measured using image analysis software (ImageJ, US National Institutes of Health, Bethesda, MD, USA).

### 4.5. Measurement of Hepatic TC and TG contents

At the end of the experiment, the liver was collected, homogenized, and lysed in ice-cold lysis buffer (PROPREP protein extraction solution, Seoul, Korea). Lipid was extracted by centrifugation at 3000 rpm for 5 min to obtain the total hepatic lipid contents. The level of hepatic TC and TG contents in the liver extraction was measured.

### 4.6. Fat Composition

At the end of the experiment, all mice were anesthetized with isoflurane and body fat profile was measured using a micro-CT system (Siemens AG, Munich, Germany). The mice were immediately placed into the micro-CT scanner bed in a prone position and scanned for 5 min. The scan was operated using the followed settings: X-ray source voltage, 55 kVp; current, 400 μA; and a 1-mm-thick aluminum filter to reduce the beam hardening artifact. The body fat volume was evaluated using image analysis software (Siemens AG, Munich, Germany). The presence of adipose tissue is indicated by the peak which is specified by the range of the thresholds in the reconstructed image. The sum of all voxel volumes between low and high threshold values was calculated to measure the visceral and subcutaneous fat.

### 4.7. Western Blot Analysis

At the end of the experiment, liver tissue was collected, homogenized, and the liver tissue lysate was centrifuged at 16,600× *g* for 15 min at 4 °C to separate total protein. The concentration of total protein was quantified using the Bradford method. The proteins were separated on a 10% sodium dodecyl sulfate-polyacrylamide gel and transferred onto a nitrocellulose membrane (Whatman, St. Louis, MO, USA). The membrane was blocked with 5% bovine serum albumin in tris-buffered saline with 0.1% Tween 20 (TBST) for 1 h and incubated at 4 °C overnight with primary antibodies against PPAR-γ, C/EBP-α (1:1000; Cell Signaling Technology, Danvers, MA, USA), and β-actin (1:1000; Cell Signaling Technology). The membrane was washed with TBST and incubated with horseradish-peroxidase-conjugated secondary antibodies (1:5000; Santa Cruz Biotechnology, CA, USA) at room temperature for 1 h. The proteins were enhanced and detected with SuperSignal^®^ West Pico chemiluminescent substrate (Thermo Scientific) using a molecular imaging system (Amersharm image 600, GE Healthcare Life Sciences, Chicago, IL, USA).

### 4.8. Statistical Analysis

Statistical analysis was performed using the Student’s *t*-test (Microsoft, WA, USA). All data are expressed as mean ± standard deviation. The statistical significance of the values was compared with ND group or HCD group. 

## Figures and Tables

**Table 1:** Composition of the 70% carbohydrate diet.

Component	g/kg	Component	g/kg
Casein	200.0	Cellulose	9.89
DL-Methionine	3.0	Vitamin Mix	10.0
Sucrose	645.6	Choline Bitartrate	2.5
Corn Starch	20.0	TBHQ, antioxidant	0.01
Maltodextrin	20.0	Mineral Mix, AIN-93G-MX (94046)	32.0
Soybean Oil	50.0	Calcium Phosphate, dibasic	4.0

**Table 2:** Effect of ethanol extract of *Amomum tsao-ko* (EAT) on body-weight change in a high-carbohydrate diet (HCD)-induced C57BL/6 mouse model.

	**1 Week**	**2 Weeks**	**3 Weeks**	**4 Weeks**	**5 Weeks**	**6 Weeks**
ND	20.53 ± 1.13	21.34 ± 1.52	22.24 ± 1.79	23.34 ± 1.58	24.09 ± 1.55	24.82 ± 1.64
HCD	20.86 ± 0.71	21.03 ± 0.80	22.03 ± 0.82	22.62 ± 0.94	23.48 ± 0.99	24.67 ± 1.63
EAT 100	20.43 ± 0.53	21.24 ± 0.70	2176 ± 0.59	22.41 ± 0.84	22.99 ± 0.91	24.30 ± 1.06
EAT 200	19.89 ± 1.20	21.25 ± 0.88	21.39 ± 0.76	22.04 ± 0.79	22.69 ± 0.84	23.90 ± 0.74
EAT 400	19.98 ± 1.19	21.76 ± 1.17	21.90 ± 0.85	22.57 ± 1.06	23.08 ± 0.87	24.27 ± 1.30
	**7 Weeks**	**8 Weeks**	**9 Weeks**	**10 Weeks**	**11 Weeks**	**12 Weeks**
ND	25.73 ± 1.51	26.16 ± 1.61	27.44 ± 1.75	27.34 ± 1.54	28.08 ± 1.82	28.19 ± 1.74
HCD	25.79 ± 1.78	26.78 ± 1.88	27.90 ± 2.12	27.91 ± 2.13	28.86 ± 2.00	30.16 ± 2.39
EAT 100	25.32 ± 0.78	25.09 ± 0.89	25.89 ± 1.26	26.23 ± 1.53	26.76 ± 1.40	27.58 ± 1.63
EAT 200	24.51 ± 1.20	24.36 ± 0.95	25.11 ± 0.95	25.89 ± 0.78	26.33 ± 1.13	26.82 ± 1.16
EAT 400	25.13 ± 1.42	25.66 ± 1.76	26.30 ± 1.86	26.51 ± 1.51	27.04 ± 1.69	27.58 ± 2.05

**Table 3:** Food intake of normal diet (ND) and HCD groups in an HCD-induced C57BL/6 mouse model.

	**1 Week**	**2 Weeks**	**3 Weeks**	**4 Weeks**	**5 Weeks**	**6 Weeks**
ND	3.24	3.18	3.54	3.32	3.32	3.47
HCD	2.63	2.89	2.56	2.58	2.79	2.96
EAT 100	2.59	2.50	2.33	2.25	2.26	2.49
EAT 200	2.28	2.80	2.37	2.45	2.44	2.45
EAT 400	2.26	2.71	2.43	2.34	2.38	2.58
	**7 Weeks**	**8 Weeks**	**9 Weeks**	**10 Weeks**	**11 Weeks**	**12 Weeks**
ND	3.46	3.05	2.84	3.20	3.25	3.61
HCD	2.62	2.72	2.30	2.66	2.67	3.16
EAT 100	2.42	2.30	2.52	2.50	2.47	3.10
EAT 200	2.38	2.45	2.25	2.20	2.49	3.20
EAT 400	2.34	2.30	2.21	2.59	2.39	3.00

## Data Availability

Data is contained within the article or supplementary material.

## Supplementary Materials

The following are available online, Figure S1: Effect of EAT on insulin level and glucose tolerance test in HCD-induced mice model.
