# Supplementary material for: Improvement of Obesity and Dyslipidemic Activity of Amomum tsao-ko in C57BL/6 Mice Fed a High-Carbohydrate Diet
Source: Molecules. 2021 Mar 15;26(6):1638. doi: 10.3390/molecules26061638 (PMC7998585; doi:10.3390/molecules26061638)
Supplement: Supplementary file 1 [file molecules-26-01638-s001.zip › molecules-1096780-supplementary.pdf]

molecules

MDPI

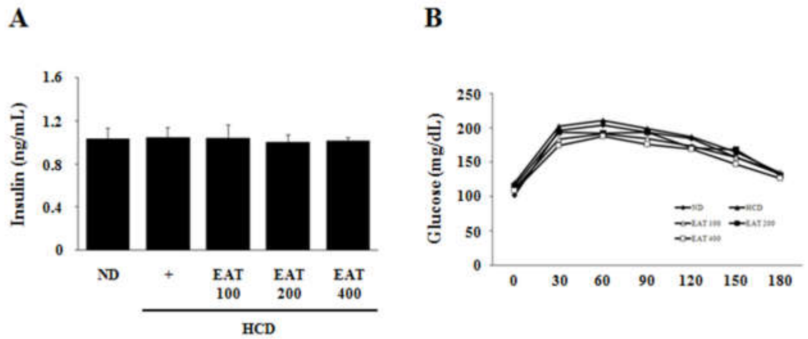

Figure S1. Effect of EAT on insulin level and glucose tolerance test in HCD-induced mice model. (A) At the end of the experiment, blood was collected from the abdominal vena cava and immediately centrifuged. Insulin level was determined using a mouse insulin ELISA kit. (B) After 12 h of fasting, a glucose solution was injected intraperitoneally (2 g/kg), and blood was collected from the tail vein at 0, 30, 60, 90, 120, 150, and 180 min. Blood glucose levels were measured using a glucometer.
